# A Computational Framework for Pattern Detection on Unaligned Sequences: An Application on SARS-CoV-2 Data

**DOI:** 10.3389/fgene.2021.618170

**Published:** 2021-05-28

**Authors:** Nikolaos Pechlivanis, Anastasios Togkousidis, Maria Tsagiopoulou, Stefanos Sgardelis, Ilias Kappas, Fotis Psomopoulos

**Affiliations:** ^1^Institute of Applied Biosciences, Centre for Research and Technology Hellas, Thessaloniki, Greece; ^2^Department of Ecology, School of Biology, Aristotle University of Thessaloniki, Thessaloniki, Greece; ^3^Department of Genetics, Development and Molecular Biology, School of Biology, Aristotle University of Thessaloniki, Thessaloniki, Greece

**Keywords:** *k*-mers, unsupervised learning, phylogenetics, feature selection, SARS-CoV-2

## Abstract

The exponential growth of genome sequences available has spurred research on pattern detection with the aim of extracting evolutionary signal. Traditional approaches, such as multiple sequence alignment, rely on positional homology in order to reconstruct the phylogenetic history of taxa. Yet, mining information from the plethora of biological data and delineating species on a genetic basis, still proves to be an extremely difficult problem to consider. Multiple algorithms and techniques have been developed in order to approach the problem multidimensionally. Here, we propose a computational framework for identifying potentially meaningful features based on *k*-mers retrieved from unaligned sequence data. Specifically, we have developed a process which makes use of unsupervised learning techniques in order to identify characteristic *k*-mers of the input dataset across a range of different *k*-values and within a reasonable time frame. We use these *k*-mers as features for clustering the input sequences and identifying differences between the distributions of *k*-mers across the dataset. The developed algorithm is part of an innovative and much promising approach both to the problem of grouping sequence data based on their inherent characteristic features, as well as for the study of changes in the distributions of *k*-mers, as the *k*-value is fluctuating within a range of values. Our framework is fully developed in Python language as an open source software licensed under the MIT License, and is freely available at https://github.com/BiodataAnalysisGroup/kmerAnalyzer.

## Introduction

During the last decade DNA sequencing technology has been revolutionized, as the advent of Next Generation Sequencing (NGS) (Castro et al., 2020) led to the production of great amounts of biological data. To this end, the analysis of genomic sequences has expanded tremendously both in scope and with respect to novel analytical methods. A research topic which recently has attracted a great deal of attention is the development of alignment-free methods and approaches, mainly for use in phylogenetic inference (Zielezinski et al., 2017; Ren et al., 2018; Bernard et al., 2019). A part of this broader family of methods is *k*-mer based analysis. This type of analysis has been used in several studies for the comparison and analysis of DNA sequences (Murray et al., 2017; Sievers et al., 2017). Alignment-based methods, such as the well-known Basic Local Alignment Search Tool (BLAST), consider the exact position and quality of similarity of every part of the sequence within the dataset. In contrast, *k*-mer estimation methods only interpret the sequences as a group of characters, therefore neglecting any positional information (Brendel et al., 1986). One of the main advantages of this kind of approach is shorter computation times in relation to sequence length (Chan and Ragan, 2013). In addition, most of the time, prior knowledge of the underlying genome sequences is not a requirement. To this end, Murray et al. (2017) have proposed a new method for a *k*-mer-based sequence comparison to estimate genetic relatedness from sequence data. Apart from sequence similarity, some studies have focused on identifying functional and evolutionary features based on *k*-mer extraction methods (Sievers et al., 2017). Results so far suggest that specific genomic regions that are recognized as evolutionary conserved by alignment-based methods, maintain local *k*-mer structures which can be identified by *k*-mer based approaches. Given the overwhelming quantities of available sequence data, a question that arises is how to identify key features across sequences that they would serve as proxies for significant phenotypic differences, aiding in this way the inference of the underlying evolutionary relationships.

In this context, a conducive and topical field of application of this kind of computational methods is the coronavirus (SARS-CoV-2) case. Nearly a year after the first report of SARS-CoV-2 in Wuhan, China, the virus has spread with an unprecedented pace causing a global pandemic. During this short time, several SARS-CoV-2 related resources have become publicly available, with the National Center for Biotechnology Information (NCBI) reaching 28,058 nucleotide records from more than 18,792 genomes^1^. Against the backdrop of recent intense research in various aspects of the coronavirus pandemic a lot of effort has been channeled towards tracking down the virus origins. Past studies were able to identify coronaviruses in several avian (Ismail et al., 2003; Cavanagh, 2007) and mammalian hosts (e.g., mice, dogs, cats) (Su et al., 2016). Amongst them, there are some pathogenic to humans that led to past epidemics. In November, 2002 a severe acute respiratory syndrome coronavirus (SARS-CoV) emerged in Guangdong, China and caused more than 8,000 infections and 774 deaths (Peiris et al., 2004), while in 2012 a Middle East respiratory syndrome coronavirus (MERS-CoV) was reported in Saudi Arabia and resulted in more than 850 deaths (Zaki et al., 2012).

With the emergence of the new SARS-CoV-2, enormous effort has taken place in order to construct an evolutionary roadmap of the virus. Full genome sequence analysis revealed that SARS-CoV-2 belongs to the betacoronaviruses, while further comparison with a bat SARS-related coronavirus (RaTG13) showed 96.7% genomic similarity (Lu et al., 2020). Later comparison to the coronavirus coming from two Malayan pangolin genomes identified a specific genomic region (spike protein), as closely related between the two coronaviruses (Xiao et al., 2020).

Here, we report on a new alignment-free method capable of processing and counting *k*-mers in a reasonable time, while evaluating multiple values of the *k* parameter concurrently. Our approach was tested on SARS-CoV-2 genomes available at https://www.ncbi.nlm.nih.gov/sars-cov-2/ in order to identify *k*-mers at the nucleotide level, from which we were able to construct an evolutionary tree. Further integration with population demographic and chronological metadata led to the identification of unique clusters and time correlated features amongst the available sequences and *k*-mers. Our results could be beneficial for a better understanding of the genetic diversity of SARS-CoV-2 and eventually help to design a robust therapeutic strategy.

## Materials and Methods

In order to investigate multiple values of the k parameter and identify a useful set of *k*-mers that are representative of the input data, our proposed algorithm is based on evaluation trees. Evaluation trees are generic, non-binary trees; in our case, each node corresponds to a single nucleotide and the full path from root to leaf corresponds to a single *k*-mer that has been located.

At the beginning of the construction of an evaluation tree, a complete tree is being produced until a depth of four. This is chosen because *k*-mers of length 1 or 2 have no meaningful value, while *k*-mers of length 3 correspond to amino acids, the distribution of which has already been investigated (Brooks et al., 2002). The main idea of the algorithm is that, for every new *k*-value that is being investigated, a new depth nodes are added to the tree that correspond to the *k*-mers within the dataset, and the procedure is divided into two parts, training and pruning. During the training phase, every node, that corresponds to a *k*-mer that has been found, is added to the tree and an evaluation method is applied in order to get a metric of how representative each node (and consequently each *k*-mer) is for the whole data set. During the pruning phase, *k*-mers with small evaluation scores are being cut off the tree. Taking all these into account, we define a tree structure *T* as a pair (*V*,*E*) where *V* corresponds to a set of nodes and *E* corresponds to a set of edges. Consequently, *E* is subset of *V* meaning that *E* = (*u*,*v*), where *u*,*v* ∈ *V*; *u* is defined as a parent and *v* is a child node.

The final tree that is being generated contains all *k*-mers that were identified by the algorithm. The root of the tree is an empty node (meaning that it doesn’t have an associated nucleotide to it), while every other node has a maximum of four children (corresponding to the four nucleotides that can be found).

**FIGURE 1:**
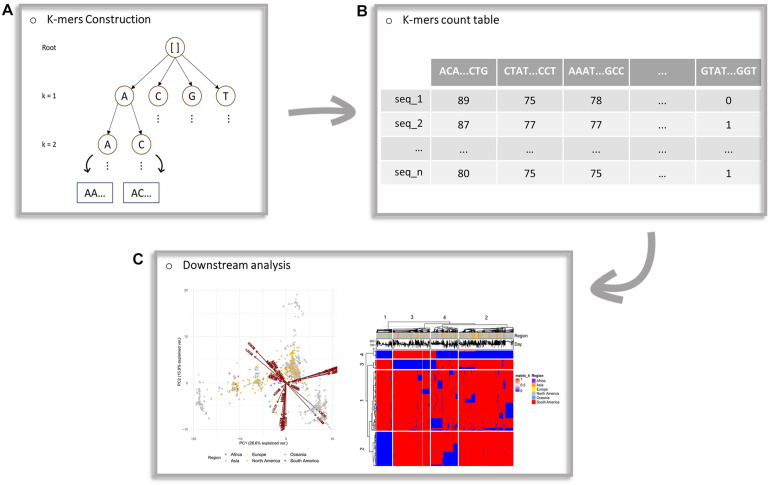
Schematic workflow of the proposed framework. **(A)** Construction of an evaluation tree. **(B)** The generated count table, with *k*-mers of multiple lengths as features (columns) and sequences as observations (rows). Each cell of the table corresponds to the number of occurrences of a *k*-mer to a particular sequence. **(C)** Principal components analysis projection on a two-dimensional space and the heatmap generated from downstream analysis of the algorithm’s output.

The current implementation of the algorithm works in a serial mode, starting with lower values of *k* and proceeding to higher values, given the user’s inputs. The maximum *k*-value is an input parameter and a variable for further investigation. When the analysis for a particular *k*-value is completed the analysis continues with the next *k*-value. In Figure 1, a schematic representation of the proposed framework is provided.

### Training

During the training phase of a particular *k*-value, a new level of depth *k* is added to the tree. The main goal is to identify all *k*-mers, a pair of (*V*,*E*), that are found in the first subset of within the dataset for the given depth. Once a *k*-mer is found, the tree is parsed to identify if the particular *k*-mer already exists in the tree. If it does, the *k*-mer count value is updated and the process continues. If not, a new node is added to the corresponding path.

The *k*-mer reading begins from the root of the tree and follows the unique path that corresponds to each specific *k*-mer. For example, when reading *k*-mer ACGT, the tree traversal begins from the root to the first level node A, then to the second level node C and so on. It is important to notice that the construction of a new level of depth *k* is being done based on the already existing structure, formed by lower *k*-values. For instance, when reading the *k*-mer ACACACGT, the main goal of the procedure is to traverse the tree from the root to node G at depth seven and create a new T node, in case it doesn’t exist. However, this scenario is not feasible if the current path has been pruned at an earlier stage at depth five. In this case, the procedure moves on to the next *k*-mer.

For every node that is added to the tree, an evaluation score is assigned to the specific node indicating the significance of the underlying *k*-mer. When the new tree is generated, the process continues with the pruning phase.

### Pruning

The pruning phase takes place at the end of the training process, when all sequences have been examined. During this process, *k*-mers - of fixed length, specified by the *k*-value that is being examined – are being re-evaluated, based on their total counts and length. If the evaluation score of a child node (*v*) is less than the score of the parent (*u*), then the particular child is cut off the tree. Once a node is removed, it cannot be reconstructed, and the related *k*-mer stops being investigated in the data set:

If evaluation_*v*_ < evaluation_*u*_, then *v* is removed from the tree.

### *k*-mer Node Evaluation

An important part of the proposed algorithm is evaluation. Every new leaf that is added to the tree is assigned with an evaluation score which represents how useful the *k*-mer, which is constructed from the related path, is to the whole data set.

The score for a particular node *n* with a depth *k*, is calculated as:

$${\text{evaluation}}_{n}=\frac{P_{k\text{mer}}}{P_{\text{uni}}}$$

where *P*_*k*mer_ represents the probability of finding the specific *k*-mer, constructed by the path root to node in the dataset, and *P*_uni_ is the probability of finding the specific *k*-mer assuming that all the other *k*-mers follow the uniform distribution. Given the latter, we have that for the fixed depth *k*:

$$P_{\text{uni}}={\left(\frac{1}{4}\right)}^{k}$$

*P*_*k*mer_ is calculated by counting the number of occurrences of a *k*-mer (count) and the whole number of the processed data (examined). Taking all into account, the evaluation is calculated as follows:

$${\text{evauation}}_{n}=4^{k}\cdot \frac{\text{count}}{\text{examined}}$$

For simplicity, the logarithmic value of the above score is used.

### *k*-mers as Data Tables

The algorithm uses as an input a *fasta* file, which contains all the unaligned DNA sequences that are going to be analyzed. After the whole range of the input *k*-values is investigated, the algorithm produces a table with the left *k*-mers as features (columns) and the sequences as observations (rows). Each column contains the number of occurrences of the related *k*-mer in every sequence. A sample output table is presented in Table 1.

**TABLE 1:** Example of the output count table produced by the algorithm.

	AACA…CTG	CTAAT…CCT	AATAT…GCC	…	GTAAT…GGT
seq_1	89	75	78	…	0
seq_2	87	77	77	…	1
…	…	…	…	…	…
seq_n	80	75	75	…	1

*Sequences are stored as rows and k-mers as columns. Each column reports how many times the related k-mer was found in every sequence.*

### Downstream Analysis

In order to identify unique observations and clusters, we further proceeded in the analysis of the algorithm’s result in *R*. Firstly, we filtered the output table by extracting all sequences that contained more than six *As* in a row or at least one *N*. The choice of filtering the polyA k-mers was driven by the fact that the reference sequence for SARS-CoV-2 (NC_045512) contains a polyA tail at the 3′-UTR region, a common event on SARS-CoV-2 sequences. As such, in order to omit the construction of k-mers based on this region, as they will not reveal any significant association, any k-mer sequences containing six As in a row or more were ultimately removed. Sequences with no metadata were also removed. Similar filtering was also used for the *k*-mers, where features with more than eight *As* in a row were removed. In addition, we applied featured selection methods in order to exclude *k*-mers with similar representation across all sequences. For this reason, we used the *nearZeroVar* function from *caret* package (Kuhn et al., 2020), which identifies and removes near zero-variance features. It should be noted that there can be instances where the data generating mechanism can create predictors (features) that only have a single unique value (i.e. a “zero-variance predictor”). For many models this may cause the model to crash or the fit to be unstable. Similarly, predictors might have only a handful of unique values that occur with very low frequencies. The concern here is that these predictors may become zero-variance predictors when the data are split into cross-validation/bootstrap sub-samples or that a few samples may have an undue influence on the model. These “near-zero-variance” predictors may need to be identified and eliminated prior to modeling. To identify these types of predictors, the following two metrics (parameters) are calculated:

• the frequency of the most prevalent value over the second most frequent value (called the “frequency ratio”), which would be near one for well-behaved predictors and very large for highly unbalanced data and
• the “percent of unique values” is the number of unique values divided by the total number of samples (times 100) that approaches zero as the granularity of the data increases.

If the frequency ratio is greater than a pre-specified threshold and the unique value percentage is less than a threshold, we might consider a predictor to be near zero-variance and therefore removed.

The remaining table was used as input to unsupervised learning techniques. More specifically, we applied PCA (*prcomp* function) and hierarchical clustering (*hclust* function, methods *Eucledian*, and *ward.D*) in order to identify unique observations or clusters that are formed in our dataset. What is more, in order to evaluate the algorithm’s integrity, we built a phylogenetic tree from the raw sequences and compared it with the dendrograms constructed by the algorithm’s output. For the alignment of the raw sequences, we used the software package *Clustal Omega* (Sievers et al., 2011) with default parameters and built the phylogenetic tree in *R* by applying neighbor joining (ape (Paradis and Schliep, 2019) package R) on a Log-Det distance matrix (Lake, 1994; Lockhart et al., 1994).

## Results

### Input Data

A total of 12,474 sequences of SARS-CoV-2 were retrieved from NCBI^2^, along with a metadata JSON file containing chronological information (i.e., collectionDate, releaseDate, and updateDate), demographic information (i.e., city, country, and continent) and information related to the collection source (cell, lung, and brain etc.). Sequences ranged in length from 28,000 to 30,000 bp.

We investigated different ranges of *k*-values for the same sequences and produced different *k*-mer count tables. From the raw count tables, we continued by filtering both sequences and features the way we have described above and ended up with 8,693 sequences and different numbers of features, depending on the data table that was analyzed.

### Comparison of Different *k*-Value Ranges

The proposed method was used to study how different *k*-mer lengths could affect sequence clustering. In order to do that, we firstly investigated lower *k*-mer sizes and therefore we started with a range of *k* ∈ [4,5]. The resulting *k*-mer counts table included 224 features. After the application of the feature selection method, a total of 148 features were retained. We proceeded with exploring a range of *k* ∈ [4,10] which resulted originally in 3464 features and later after the application of the selected criterions in 45 features. Finally, we investigated higher ranges of *k*-values, considering the length of the raw sequences. For that, we chose a range of *k* ∈ [4,80] which included 3861 features before the filtering process and 236 *k*-mers after the filtering process.

**FIGURE 2:**
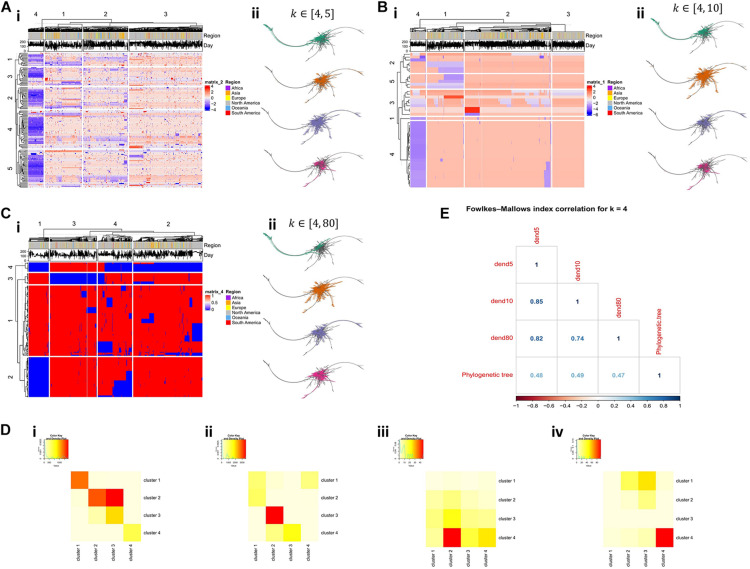
Comparison of results produced by the investigation of multiple *k*-ranges. **(Ai)** Heatmap of produced features in the range of *k* ∈ [4,5] and **(Aii)** the projection of the underlying sequence clusters in the phylogenetic tree produced by the raw sequences. **(Bi, ii)** Heatmap and the projected sequence clusters on the phylogenetic tree for *k* ∈ [4,10]. **(Ci, ii)** Heatmap and the projected sequence clusters on the phylogenetic tree for *k* ∈ [4,80]. **(Di)** Overlap between sequence clusters for *k* ∈ [4,5] (depicted as columns) and *k*-mer clusters (depicted as rows) for *k* ∈ [4,10]. **(Dii)** Overlap between sequence clusters for *k* ∈ [4,10] (depicted as columns) and *k*-mer clusters (depicted as rows) for *k* ∈ [4,80]. **(Diii)** Overlap between sequence clusters for *k* ∈ [4,5] (depicted as columns) and *k*-mer clusters (depicted as rows) for *k* ∈ [4,10]. **(Div)** Overlap between sequence clusters for *k* ∈ [4,10] (depicted as columns) and *k*-mer clusters (depicted as rows) for *k* ∈ [4,80]. **(E)** Correlations based on the Fowlkes-Mallows index with 4 clusters, between the three sequence dendrograms (generated for the three ranges) and the phylogenetic tree.

Hierarchical clustering was performed on all final tables that remained from the filtering process, depicting features (i.e., *k*-mers) as rows and sequences as columns (Figures 2Ai–Ci). In addition, we applied hierarchical clustering both on features and sequences, using as a distance measure the Euclidean distance. We chose to assemble both sequences and *k*-mers into 4 groups and searched for overlapping clusters between the sequence clusters (Figures 2Di,Eii) and the feature clusters (Figures 2Diii, Eiv) for the three ranges. We proceeded by creating a phylogenetic tree from the raw aligned sequences and projecting the resulting sequence clusters on the phylogenetic tree (Figures 2Aii–Cii). Finally, the Fowlkes–Mallows index (Fowlkes and Mallows, 1983; Wallace, 1983) was used to determine the similarity between the underlying distances in the three dendrograms produced by the algorithm and the dendrogram that was derived from the phylogenetic analysis (Figure 2E).

Dendrograms produced by the ranges *k* ∈ [4,5] and *k* ∈ [4,10] show higher correlation scores between them, as opposed to the dendrogram produced by *k* ∈ [4,80] (Figure 2E). Moreover, we observe that there is no strong similarity between the distances produced by the different *k*-mer based trees and the phylogenetic tree, as all three *k*-mer based trees exhibit a Fowlkes-Mallows correlation of 0.5 to the distances of the phylogenetic tree. However, given the known difficulty (Morel et al., 2020) in producing a valid phylogenetic tree for these particular sequences (Vasilarou et al., 2020), this moderate correlation level is an encouraging result.

### Investigation of 80-mers

We continued our analysis by focusing only on 80-mers, as they seem to preserve a clearer distinction of the sequence grouping. Unsupervised principal components analysis (PCA) was applied on the table derived from the feature selection step (*n* = 236).

**FIGURE 3:**
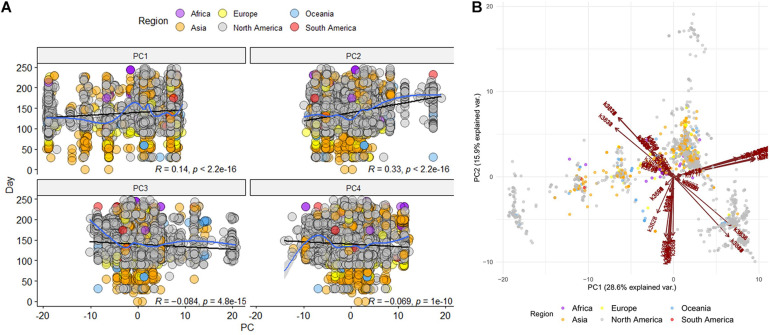
Unsupervised Principal Components Analysis of the selected sequences (*n* = 236) **(A)** Time correlation for every principal component (PC) **(B)** Projection of all raw sequences on the PC1 – PC2 space along with their demographic meta data. Arrows correspond to the *k*-mers that are projected on PC1 and PC2.

The first 10 principal components (i.e., PCs) gather almost 90% of explained variance of the dataset, with PC1 describing 28.6% of the dataset and PC2 almost 16%. In addition, each sequence along with its demographic information is projected on the two-dimensional PC1-PC2 space, where distinct groups of sequences seem to be formed (Figure 3B). Furthermore, the time evolution of the sequences in relation to every principal component is investigated (Figure 3A), where a time correlation of PC2 is revealed. The PC2 showed a statistically significant correlation with the day of sampling (Pearson *R* = 0.33,*p* < 0.001); in our study, we considered Dec 1 2019 as the first day (day 0) of the sampling.

**FIGURE 4:**
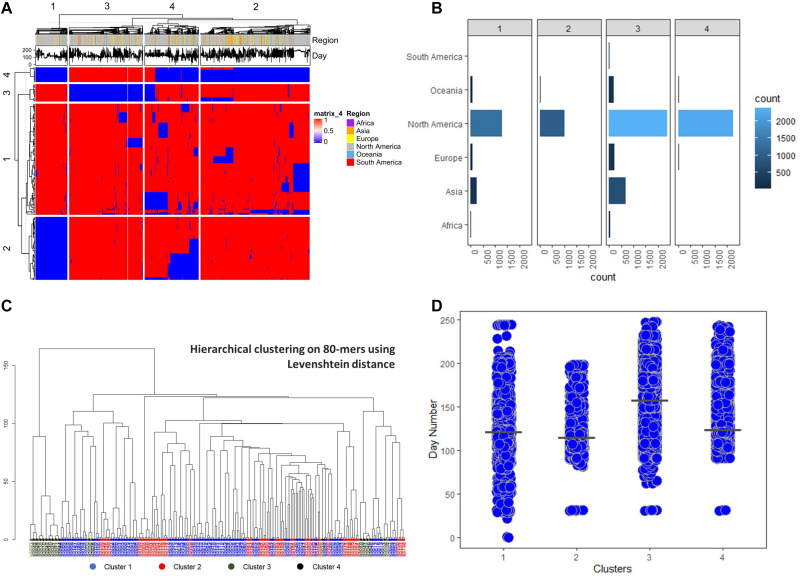
Hierarchical clustering analysis of the 236 selected sequences features **(A)** The generated heatmap for *k* ∈ [4,80] **(B)** Number of sequences for every region in the sequence clusters. **(C)** Hierarchical clustering of the 236 80-mers based on Levenshtein distance. **(D)** Day count of the sequences in relation to the obtained clusters, considering December 1, 2019 as day 0.

Having a general perspective of the grouping between sequences, we proceeded with hierarchical clustering with 4 groups both on features and observations. The resulting clustering is visualized in a heatmap (Figure 4A) and it depicts a unique signature of *k*-mer clusters, able to clearly define the sequence groups. We calculated the Levenshtein distance across all 80-mers in order to identify additions or substitutions between the *k*-mers on the nucleotide level (Figure 4B). We observed that *k*-mer clusters 3 and 4, which seem to follow a complementary distribution, are relatively close to each other, implying few nucleotide changes compared with the other groups. Finally, the obtained sequence groups were annotated with their corresponding chronological and demographic metadata (Figures 4C,D), where the resulting clusters show significant time correlated differences.

### Performance Evaluation

We used a 24-core Unix cluster with 220G RAM for the alignment of raw sequences and the testing of the proposed algorithm. The main *k*-mer detection tool is written in Python language and is freely available at https://github.com/BiodataAnalysisGroup/kmerAnalyzer. The downstream analysis was performed in *R* and the source code is located in the subfolder *R* of the provided repository.

Clustal Omega (Sievers et al., 2011) required approximately 18 days to generate a multiple sequence alignment using the default parameters. In addition, an extra day was required to build a distance matrix to be used for phylogenetic tree inference. The proposed framework was executed with different *k*-mer length ranges and as independent runs on the same computational infrastructure. The corresponding execution times are given in Table 2.

**TABLE 2:** Execution times of the proposed algorithm.

Range	Duration
*k* ∈ [4,5]	400 min (6 h, 40′)
*k* ∈ [4,10]	1236 min (20 h, 30′)
*k* ∈ [4,80]	17635 min (12 d, 6 h)

## Discussion

Next generation sequencing has revolutionized the generation of data. An emerging task has been the identification of possible features for sample data classification. For this reason, reference sequence databases are often used, and new methods, for managing the great amount of data, are being developed (Allesøe et al., 2020). In this study, we propose a new computational framework for *k*-mer feature identification from unaligned sequences by investigating multiple values of *the k* parameter. The algorithm produces a *k*-mer count table which can be used for the extraction of key features capable of clustering the raw sequences.

In order to evaluate our methods, we applied the proposed tool on SARS-CoV-2 sequences from the NCBI and studied three different ranges; *k* ∈ [4,5], ***k* ∈ [4,10]**, and *k* ∈ [4,80]. We managed to cluster the raw sequences into 4 groups and compared the tool’s output with the traditional approach involving multiple sequence alignment, using Clustal Omega, and phylogenetic tree inference. Comparison of execution times between the two methodologies suggest that, for the same input dataset, our method can produce a count table much faster than an alignment by Clustal Omega. Further investigation of the dendrograms generated by the three experiments we performed and the phylogenetic tree generated by the raw input sequences, showed a moderate correlation. This is a very unexpected result, especially if we take into consideration the growing concern regarding the reliability of SARS-CoV-2 generated phylogenies. Recent studies (Morel et al., 2020; Vasilarou et al., 2020) support that the relatively high nucleotide substitution rate expected for viruses, along with high recombination rates, imply crucial difficulties that need to be overcome in order to generate valuable conclusions.

We further proceeded in analyzing the output count table of 80-mers. We were able to identify a unique signature of a group of *k*-mers which cluster the input sequences. Moreover, we integrated our results with chronological metadata and found a correlation between the second principal component (PC2) and the time that has passed since the first incident, which implies a set of *k*-mers that play a vital role for the virus evolution.

Moving beyond the constraints of the use case presented here, the proposed methodology can be readily applied to a wider range of applications that depend on sequence data. The identified k-mers define a novel feature space that can be directly leveraged towards the application of machine learning approaches. As an example, supervised machine learning can be applied to classify sequence data using only k-mers as input-features. It is also important to highlight that there is no particular constraint to the sequence length; the method is equally applicable to complete sequences as well as short reads generated through NGS. In this context, there is a wider range of applications, including metagenomics, virtual barcoding and overall alignment-free sequence data processing.

Although the proposed method can provide a profound representation of the analyzed sequences, there are also some limitations. A key element of the proposed algorithm is the evaluation function. In our case, the evaluation of every node, and every *k*-mer, is based on probabilities of finding specific *k*-mers. As this is a first implementation of the proposed methodology it is worth investigating different evaluation functions which, consequently, may result to different sets of *k*-mers. What is more, most of the sequences of the input dataset were from North America, thus making it impossible to draw a firm conclusion on the overall picture of the evolution of SARS-CoV-2. An application on a more diverse dataset, not only on SARS-CoV-2 but also on other genomes, would give a clearer view of the benefits of the framework.

It is worth mentioning that the optimization of the sub-procedures and functions of the proposed method is an open task for further investigation and will be considered in future work. Moreover, a next step would be to facilitate a complementary approach that will apply an exhaustive search (brute force) of all possible *k*-mers that can be retrieved from the raw sequence data. This approach may result in a more meaningful count table which would contain all possible *k*-mers present in the input dataset, and therefore increase the possibility of identifying a strong underlying signal.

Finally, the proposed framework has been implemented as an open-source, fully documented tool, available on GitHub and easily applicable to any type of sequence data. It provides a new perspective towards sequence analysis, by offering comparably short execution times and meaningful representation of the underlying information structure.

## Data Availability Statement

The data used in this study were retrieved from NCBI (https://www.ncbi.nlm.nih.gov/sars-cov-2/). The source code of the software that performed the analysis is available through the GitHub repository at https://github.com/BiodataAnalysisGroup/kmerAnalyzer.

## Author Contributions

AT designed the study and developed the tool. NP and MT analyzed the data, designed the study, and wrote the manuscript. SS and IK supervised the study and reviewed the submitted version. FP designed and supervised the study and reviewed the manuscript. All authors contributed to the article and approved the submitted version.

## Conflict of Interest

The authors declare that the research was conducted in the absence of any commercial or financial relationships that could be construed as a potential conflict of interest.

## Footnotes

1 https://www.ncbi.nlm.nih.gov/sars-cov-2/

2 https://www.ncbi.nlm.nih.gov/datasets/coronavirus/genomes/
